# Effects of inflammation and soluble epoxide hydrolase inhibition on oxylipin composition of very low‐density lipoproteins in isolated perfused rat livers

**DOI:** 10.14814/phy2.14480

**Published:** 2021-02-24

**Authors:** Rachel E. Walker, Olga V. Savinova, Theresa L. Pedersen, John W. Newman, Gregory C. Shearer

**Affiliations:** ^1^ Department of Nutritional Sciences The Pennsylvania State University University Park PA USA; ^2^ Department of Biomedical Sciences New York Institute of Technology College of Osteopathic Medicine Old Westbury NY USA; ^3^ Sanford Research University of South Dakota Sioux Falls SD USA; ^4^ Advanced Analytics Davis CA USA; ^5^ Department of Food Science and Technology University of California Davis CA USA; ^6^ Obesity and Metabolism Research Unit Western Human Nutrition Research Center Agricultural Research Service US Department of Agriculture Davis CA USA; ^7^ Sanford School of Medicine University of South Dakota Sioux Falls SD USA

**Keywords:** compartmental modeling, fatty acid/metabolism, lipoproteins, perfusion, polyunsaturated fatty acids, soluble epoxide hydrolase inhibitor

## Abstract

Oxylipins are metabolites of polyunsaturated fatty acids that mediate cardiovascular health by attenuation of inflammation, vascular tone, hemostasis, and thrombosis. Very low‐density lipoproteins (VLDL) contain oxylipins, but it is unknown whether the liver regulates their concentrations. In this study, we used a perfused liver model to observe the effect of inflammatory lipopolysaccharide (LPS) challenge and soluble epoxide hydrolase inhibition (sEHi) on VLDL oxylipins. A compartmental model of deuterium‐labeled linoleic acid and palmitic acid incorporation into VLDL was also developed to assess the dependence of VLDL oxylipins on fatty acid incorporation rates. LPS decreased the total fatty acid VLDL content by 30% [6%,47%], and decreased final concentration of several oxylipins by a similar amount (13‐HOTrE, 35% [4%,55%], −1.3 nM; 9(10)‐EpODE, 29% [3%,49%], −2.0 nM; 15(16)‐EpODE, 29% [2%,49%], −1.6 nM; AA‐derived diols, 32% [5%,52%], −2.4 nM; 19(20)‐DiHDPA, 31% [7%,50%], −1.0 nM). However, the EPA‐derived epoxide, 17(18)‐EpETE, was decreased by 75% [49%,88%], (−0.52 nM) with LPS, double the suppression of other oxylipins. sEHi increased final concentration of DHA epoxide, 16(17)‐EpDPE, by 99% [35%,193%], (2.0 nM). Final VLDL‐oxylipin concentrations with LPS treatment were not correlated with linoleic acid kinetics, suggesting they were independently regulated under inflammatory conditions. We conclude that the liver regulates oxylipin incorporation into VLDL, and the oxylipin content is altered by LPS challenge and by inhibition of the epoxide hydrolase pathway. This provides evidence for delivery of systemic oxylipin signals by VLDL transport.

## Graphical abstract

Appearance of linoleate label in epoxides (EpOMEs) was greater than for alcohols (HODEs).

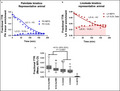

## 1 INTRODUCTION

Oxylipins are produced from polyunsaturated fatty acids (PUFA) by enzymes of the lipoxygenase (LOX) and cytochrome P450 (CYP450) families (Shearer, Harris, Pedersen, & Newman, 2010). Oxylipins mediate inflammation, cell proliferation, and inflammatory resolution, among other processes (Buczynski, Dumlao, & Dennis, 2009; Gabbs, Leng, Devassy, Monirujjaman, & Aukema, 2015; Shearer & Newman, 2009). Although the oxylipin products of arachidonic acid (AA) have been most extensively studied, products of linoleic acid (LA), α‐linolenic acid (ALA), eicosapentaenoic acid (EPA), ω‐3 docosapentaenoic acid (DPA‐ω3), and docosahexaenoic acid (DHA) also have unique functions (Gabbs et al., 2015; Markworth et al., 2016). The CYP450 epoxygenase pathway produces fatty acid epoxides from AA, epoxyeicosatrienoates (EpETrEs), which have anti‐hypertensive and anti‐inflammatory functions (Oni‐orisan et al., 2016; Spector, 2009; Spector & Norris, 2007). The DHA‐derived epoxides, epoxydocosapentaenoates (EpDPEs), have similar function to the EpETrEs, but some regioisomers are more potent (Ye et al., 2002). CYP450‐derived metabolites of ω‐3 PUFA can also suppress pathologic retinal and choroidal neovascularization (Gong et al., 2016; Shao et al., 2014; Yanai et al., 2014). The enzymes in both the LOX and CYP450 families can produce mid‐chain hydroperoxides, and then alcohols, such as the AA‐derived hydroxyeicosatetraenoates (HETEs), with additional biological activities (Shearer et al., 2010).

Oxylipins can be esterified into phospholipids or triglycerides and circulate in plasma lipoproteins to be used or stored by peripheral tissues (Shearer & Newman, 2009). In fact, the majority of oxylipins circulating in plasma are esterified in the lipoproteins (Newman, Pedersen, Brandenburg, Harris, & Shearer, 2014; Schebb et al., 2014; Shearer & Walker, 2018). Oxylipins circulating in lipoproteins can be released by lipoprotein lipase and taken up directly into cells or bind to cell surface receptors (Shearer & Newman, 2008; Wang et al., 2009), providing a potential systemic signaling role for lipoprotein oxylipins (Shearer & Newman, 2009). Very low‐density lipoproteins (VLDL), the primary carriers of plasma triglycerides, are synthesized in the liver, but it is unknown whether the liver regulates the incorporation of esterified oxylipins into the VLDL. There is evidence that VLDL particles can directly interact with endothelial cells, which can induce a pro‐inflammatory response and alter cell membrane characteristics (Magnifico et al., 2017; Wang et al., 2009; Wang, Sapuri‐Butti, Aung, Parikh, & Rutledge, 2008), contributing to vascular dysfunction. Oxylipins in VLDL could be a potential molecular explanation for the role of VLDL in inflammation.

Each lipoprotein class has its own unique profile of oxylipins (Newman, Kaysen, Hammock, and Shearer 2007; Newman et al., 2014; Proudfoot et al., 2009; Shearer et al., 2018), and VLDL are especially important transporters of epoxides, diols, and mid‐chain alcohols (Newman et al., 2007; Shearer & Newman, 2008). This specificity by lipoprotein class has been demonstrated in both animal and human models, with the HDL having the highest concentration (Newman et al., 2007, 2014), likely due to the phospholipid enrichment of this particle class. However, there is evidence that VLDL oxylipin profiles undergo important changes during disease and postprandial states (Newman et al., 2007; Wang et al., 2009). It is unknown whether hepatic inflammation alters the VLDL oxylipin profile and how these alterations could affect systemic inflammatory status.

Epoxides are a key oxylipin class of interest, since they are anti‐inflammatory, and are abundant in the VLDL particle. Biological transformation of most fatty acid epoxides is primarily accomplished through hydrolysis by soluble epoxide hydrolase (sEH)‐dependent hydrolysis to 1,2‐ or vicinal diols (Spector & Kim, 2015). Because of the beneficial effects of epoxides, sEH inhibitors have been investigated as potential targets for a wide array of diseases (Morisseau and Hammock, 2012), including cardiovascular disease (CVD) and chronic inflammatory diseases (Duflot, Roche, Lamoureux, Guerrot, & Bellien, 2014), and have shown potential in animal models to reduce atherosclerosis (Ulu et al., 2008) and nonalcoholic fatty liver disease (Wells et al., 2016). The changes in VLDL fatty acid and oxylipin composition would provide evidence that the liver plays an important role at the point of synthesis in the altered VLDL of chronic metabolic conditions. To isolate the role of the liver deuterium‐labeled fatty acids in an isolated, perfused liver model will be used.

### 1.1 Hypotheses

In this study, we aim to demonstrate a role for the liver in modifying VLDL composition by testing the following hypotheses: (a) circulating nonesterified PUFAs such as LA are precursors for VLDL‐oxylipins; (b) hepatic conversion of PUFAs to VLDL‐oxylipins is responsive to a pro‐inflammatory stimuli such as bacterial lipopolysaccharide (LPS); (c) stabilization of lipid epoxides by a sEH‐inhibitor will increase VLDL‐EpOME content; (d) that oxylipin content of VLDL is determined by the kinetics of hepatic fatty acid uptake, but this correlation is altered by LPS and sEH inhibition (sEHi). To test these hypotheses, we used a perfused rat liver model and mechanistic kinetic analysis by multi‐compartmental modeling.

## 2 MATERIALS AND METHODS

### 2.1 Animals and experimental design

Male Sprague–Dawley rats, weighing 250 g, were purchased from Charles River (*N* = 16) and kept under 12 hr light/dark cycle with ad libitum access to food and water for 4–6 weeks. One day before planned terminal experiments, rats were given an intraperitoneal injection of 10 mg/kg lipopolysaccharide (LPS) or saline and fasted overnight (18 hr). Rats were euthanized by exsanguination under a combination of ketamine (100 mg/kg) and xylazine (12.5 mg/kg) and inhaled isofluorane anesthesia (1.5%–3%) by opening the inferior vena cava. Animal use protocols were approved by the Institutional Animal Care and Use Committee (IACUC) at Sanford Research/USD, Sioux Falls, SD.

A 2 × 2 factorial design was used for treatment assignment. Eight rats were given LPS injection, and 8 rats were assigned for treatment of the livers with the sEH inhibitor, 12‐(3‐adamantan‐1‐yl‐ureido)‐dodecanoic acid (AUDA), during perfusion. Two rats were excluded from final analysis due to experimental inconsistencies: in one case, the rat was not fasted, and in the second case, the liver perfusion instrumentation failed (Control, *N* = 3; LPS, *N* = 4; AUDA, *N* = 3; LPS + AUDA, *N* = 4; Final Total, *N* = 14).

### 2.2 Liver perfusion

Livers were perfused as described previously (Shearer, Couser, & Kaysen, 2004). Briefly, rats were anesthetized and placed on a heating pad at 37°C. The abdomen was opened, exposing the liver and hepatic portal vein, which was cannulated. The inferior vena cava was nicked, and perfusion immediately begun with approximately 100 ml of DMEM (Gibco, Grand Island, NY, USA) supplemented with 1% fatty acid‐free bovine serum albumin (Sigma Chemical Co.) and 5 U/ml heparin at a flow rate of 5 ml/min. After complete removal of the blood, a recirculating perfusion system was established via the hepatic portal vein cannula, ligated vena cava, and a temperature controlled membrane oxygenating chamber (Radnoti, Thomas Scientific, Swedesboro, NJ). Two hundred and fifty milliliter of starting volume of recirculating perfusate included 20% sheep RBC (Innovative research, Novi, MI); 5% fatty acid‐free BSA; 0.5 mM Palmitic acid‐d2, (^2^H‐PA, Cambridge Isotope Laboratories); and 0.05 mM Linoleic acid‐d4 (^2^H‐LA, Cayman Chemical) in DMEM. AUDA (10 µM, Cayman Chemicals) or vehicle (0.1% ethanol) were included in the perfusate according to the experimental group assignment. Recirculating perfusion was continued for 180 min at 13 ml/min. 7.5 ml aliquots were withdrawn at 0, 7.5, 15 min and every 15 min thereafter. The remaining perfusate (approximately 150 ml) was also preserved for the final fatty acid measurements.

### 2.3 Separation of VLDL from perfusate

VLDL separation from Liver perfusate was performed as described by Edelstein and Scanu (Edelstein & Scanu, 1986). The aqueous component was isolated from erythrocytes through centrifugation at 300*g* for 10 min. Subsequently, VLDL fractions were separated by ultracentrifugation at 4°C as previously described (Shearer et al., 2004).

### 2.4 VLDL oxylipin extraction and analysis

Although it is often informative to measure esterified and nonesterified oxylipins separately, we have previously demonstrated that nonesterifed oxylipins are present in very low quantities in VLDL (Shearer and Newman, 2008). Because these samples were highly diluted VLDL fractions, the nonesterified oxylipins would be near or below our limit of detection. Therefore, only the total oxylipin concentrations will be considered for this study. Total oxylipins were isolated from VLDL fractions with a modified Smedes extraction, hydrolyzed by sodium methoxide, and isolated by solid phase extraction. They were then qualitatively analyzed by liquid chromatography, negative mode electrospray ionization, triple quadrupole mass spectrometry (LC/MS/MS) with multireaction monitoring (MRM), as previously reported (Grapov, Adams, Pedersen, Garvey, & Newman, 2012; Smedes, 1999). Oxylipins detected included epoxide, mid chain alcohol, diol, and ketone products of LA, AA, EPA, and DHA.

Briefly, 100 µl of VLDL isolate was enriched with 5 µl of methanolic 0.1 mg/ml butylated hydroxy toluene and EDTA, diluted with isopropanol, and extracted in cyclohexane using a double extraction protocol. The organic phases were combined and dried, and total lipid residues were reconstituted in 20 µl toluene and 20 µl methanol. Esterified oxylipins were transformed into free acids for LC–MS/MS analysis by trans‐methylation followed by hydrolysis by mixing the lipid extract with 100 µl 0.5 M methanolic sodium methoxide, incubating for 1 hr at 60°C, adding 100 µl of water, and incubating again at 60°C for another hour. Oxylipin free acids were then isolated with 1 cc 10 mg Oasis HLB solid phase extraction 96‐well plates (Waters Corp Inc.; Milford, MA), and eluted with 0.5 ml 1.0% acetic acid in methanol, followed by 1.0 ml ethyl acetate. Solvents were removed under vacuum, and residues were reconstituted in 50 µl 100 nM 1‐cyclohexyl ureido, 3‐dodecanoic acid (CUDA) internal standard solution.

Labeled and native oxylipins were analyzed by electrospray ionization LC‐MS/MS in previously reported methods (Agrawal, Hassoun, Foolad, Pedersen, & Sivamani, 2017). Calibration standards containing internal standards were run to verify retention times, and were purchased from Cayman Chemical (Ann Arbor, MI), Medical Isotopes (Pelham, NH), Avanti Polar Lipids Inc. (Alabaster, AL), or Larodan Fine Lipids (Malmo, Sweden). Data were processed with AB Sciex MultiQuant v. 3.0. Tracer enrichment was calculated as tracer area counts (tracer AC)/(tracer AC + tracee AC). Mass transitions of labeled oxylipins are shown in Table S1.

### 2.5 Fatty acid analysis

Fatty acid analysis of the VLDL and nonesterified fatty acid fractions were performed by modified Bligh and Dyer lipid extraction (Bligh & Dyer, 1959), fatty acid methylation, and analysis of fatty acid methyl esters by gas chromatography and mass spectrometry (GC‐MS). Briefly, lipids from 200 µl of sample were extracted in methylene chloride with an added nonesterified fatty acid surrogate mixture, containing tridecanoic acid (C13:0), nonadecanoic acid (C19:0), and docosatrienoic acid (C22:3n3). Lipid extracts were dried and incubated at 100°C for 30 min in reagent mixture including methanol, benzene, and 14% boron trifluoride in methanol (35:30:35, vol:vol:vol). Fatty acid methyl esters were extracted in hexane with internal standard, 10 µg/ml heptadecenoic acid in hexane (Morrison and Smith, 1964).

Fatty acid methyl esters were analyzed on a QC2010 GC‐MS (Shimadzu, Japan) with a SP‐2560 capillary column (Supelco, Bellefonte, PA). The mass spectrometer was run with electron‐impact ionization in scan mode, collecting 30–400 m/z data. Fatty acids detected included palmitic acid (PA; C16:0), palmitoleic acid (POA; 16:1n7), stearic acid (SA; C18:0), oleic acid (OA; C18:1n9), vaccenic acid (VA; C18:1n7), linoleic acid (LA; C18:2n6), dihomo‐γ‐linolenic acid (DGLA; C20:3n6), arachidonic acid (AA; C20:4n6), docosatetraenoic acid (DTA; C22:4n6), ω‐6 docosapentaenoic acid (DPA‐ω6; C22:5n6), ω‐3 docosapentaenoic acid (DPA‐ω3; C22:5n3), and docosahexaenoic acid (DHA; C22:6n3). Tracer analysis was done by obtaining area counts (AC) for the 76 m/z ion (tracer) and 74 m/z ion (tracee) for PA and the 70 m/z ion (tracer) and 67 m/z ion (tracee) for LA. Tracer enrichment was calculated as (tracer AC)/(tracer AC + tracee AC). All mass spectrum analysis was done using GCMSsolution software (Shimadzu, Japan).

### 2.6 Development of compartmental kinetic model

FA tracer enrichment data were used to construct kinetic models of PA and LA. A 2‐compartment model was considered first, and additional compartments were added to achieve better fit, or removed to improve convergence. Goodness of fit was assessed using the fractional standard deviation and sums of squares of the solved model. Finally, PA and LA models were linked using a coefficient parameter to decrease collinearity. Compartmental modeling was done using WinSAAM 3.3 software.

### 2.7 Statistical Analysis

Distributions of all continuous variables were evaluated for normality. The variables that were non‐normally distributed were log‐transformed when appropriate to satisfy model assumptions. ANOVA, linear regression analyses, and mixed model regressions were performed using JMPpro 13.1 (SAS Institute, Inc.) or GraphPad Prism 6 (GraphPad Software, Inc., San Diego, CA).

## 3 RESULTS

**FIGURE 1:**
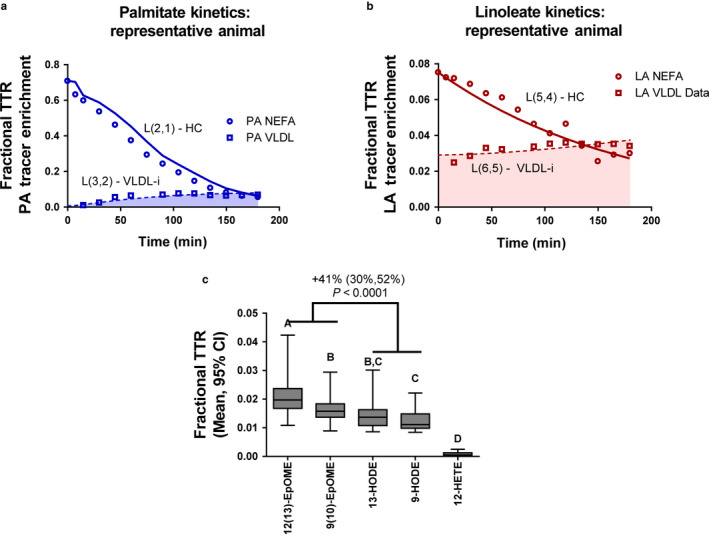
Kinetic analysis of tracer uptake. (a) Representative time‐dependent palmitic acid (PA) tracer enrichment (LPS treated rat) in nonesterified fatty acids (NEFA) and VLDL sampled from the perfusate. Lines represent model solved tracer enrichment. Nonesterified PA was included in the model as a forcing function. L(2,1) represents hepatic clearance (HC) of PA and L(3,2) represents VLDL PA incorporation rate (VLDL‐i); (b) Representative linoleic acid (LA) tracer enrichment (LPS treated rat) in nonesterified fatty acids (NEFA) and VLDL. Lines represent model solved tracer enrichment; L(5,4) represents HC of LA and L(6,5) represents LA VLDL‐i; (c) Deuterium label was detected in 4 LA‐derived oxylipins, and was highest in 12(13)‐EpOME. A small amount of label was also detected in the AA‐derived alcohol, 12‐HETE. Tracer enrichment was significantly higher in EpOMEs (0.019 [0.016,0.022]) than HODEs (0.013 [0.011,0.016]), regardless of LPS and sEHi treatments (*p* < .0001, *N* = 14 samples). Tracer enrichment was calculated as tracer to tracee ratio (TTR), or TTR = tracer area counts (AC)/(tracer AC + tracee AC). Means with different letters are significantly different by Tukey HSD post hoc analysis

The deuterium‐labeled nonesterified fatty acids (PA and LA) in the perfusate decreased over time, accompanied by a gradual increase in labeled fatty acids in VLDL (Figure 1). The relatively low tracer enrichment of VLDL fatty acids reflects the large hepatic fatty acid pool, where in addition to being packaged into VLDL, the labeled fatty acids could be stored or oxidized for energy within the liver. Fatty acid peaks were not detectable above noise at 0 and 7.5 min, and so VLDL data analysis began at 15 min. Tracer‐derived label was detected in LA‐derived hydroxyoctadecadienoates (HODEs), epoxyoctadecenoates (EpOMEs), and AA‐derived 12‐hydroxytetraenoate (12‐HETE) in VLDL isolated from the final perfusate (180 min). The final tracer enrichment measured in EpOMEs was 41% greater than that measured in HODEs. These data demonstrate that circulating nonesterified PUFAs are taken up by the liver and used as precursors for VLDL‐oxylipins.

**FIGURE 2:**
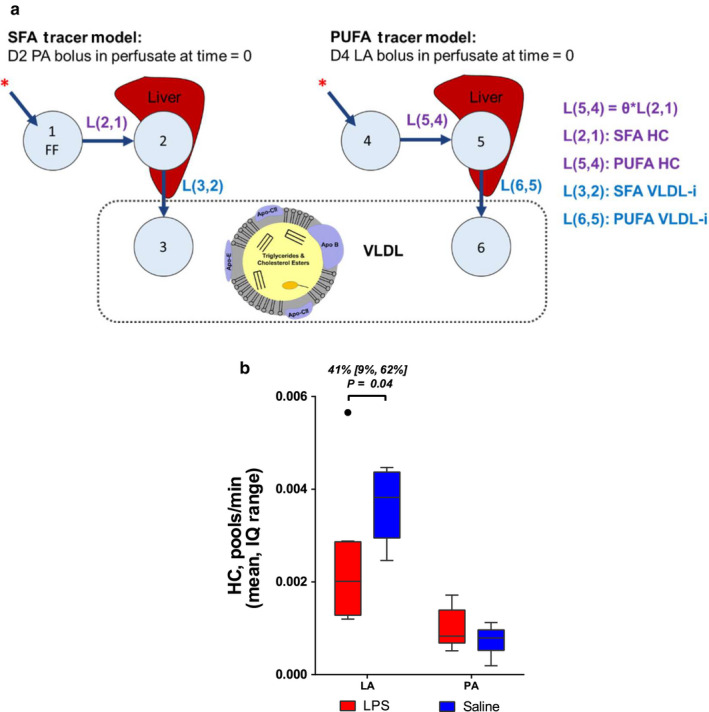
Compartmental model of saturated fatty acid (SFA) and polyunsaturated fatty acid (PUFA) hepatic kinetics. (a) The linoleic acid (LA) model was used to calculate hepatic clearance (HC) and VLDL incorporation (VLDL‐i) of PUFA. Compartment 1 and 4 represent nonesterified fatty acids in perfusate. Compartment 1 was included in the model as a forcing function (FF). Compartment 2 and 5 represent processing in the liver; and compartment 3 and 6 represent fatty acids in VLDL. (b) LPS suppresses hepatic clearance (HC) of linoleic acid (LA), but HC of palmitic acid (PA) was unchanged with treatment. Soluble epoxide hydrolase inhibition (sEHi) had no effect on HC

The final compartmental model describing PUFA hepatic clearance (HC) of fatty acids and VLDL incorporation (VLDL‐i) is shown in Figure 2a, which describes a parallel model linking hepatic PA uptake to LA uptake. In the PA portion, compartment 1 represented nonesterified fatty acids in perfusate, compartment 2 represented fatty acid processing within the liver, and compartment 3 represented fatty acids in VLDL. Kinetic parameters used to fit the model included transfer coefficients from compartment 1 to compartment 2 (L(2,1)) and from compartment 2 to compartment 3 (L(3,2)). L(2,1) represents hepatic clearance (HC) of fatty acid and L(3,2) represents VLDL fatty acid incorporation rate (VLDL‐i). A parallel model was constructed for LA, and the models were linked by the parameter, Ɵ, a coefficient that made the value of LA HC (L(5,4)) dependent on the value of PA HC (L(2,1)). Linking the models resolved collinearity between HC and VLDL‐i, improving the identifiability of the parameters. Mean HC (pools/min) was higher for LA (0.0027), compared to PA (0.00079) (*p* < .0001), but this difference should be interpreted with caution. In the PA model, a forcing function was used to fit the HC data, making direct interpretation of this value invalid by this method. There was no significant difference between PA VLDL‐i and LA VLDL‐i. An analysis of the kinetics of LA and PA uptake showed that LPS treatment decreased HC for LA, but not PA (Figure 2b).

**FIGURE 3:**
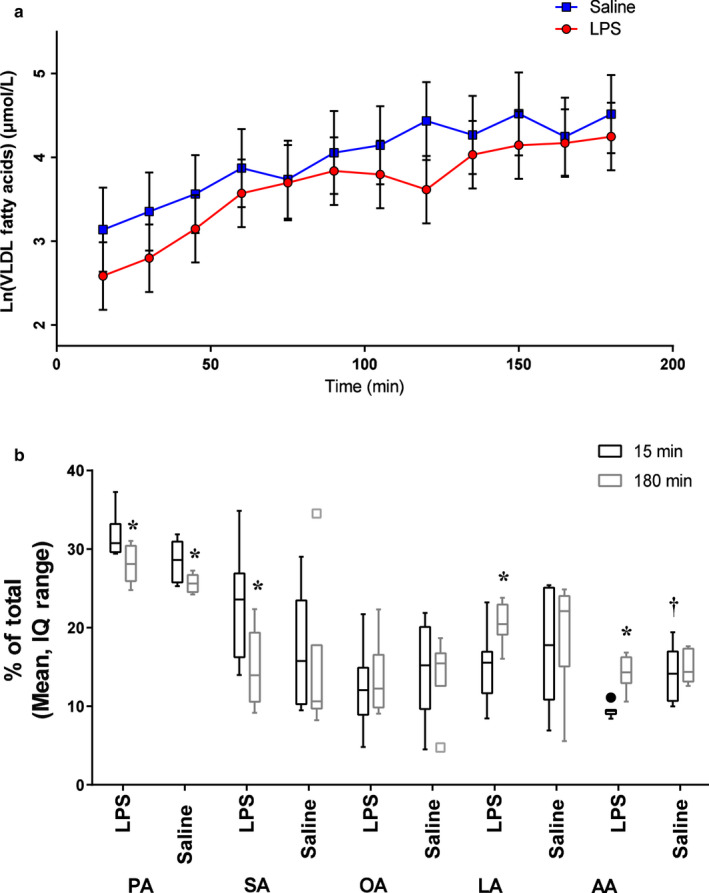
Changes in VLDL fatty acids. (a) Time‐dependent increase in total fatty acids in VLDL fractions. LPS decreased total VLDL fatty acid concentration across time points. (b) From 15 to 180 min, %PA decreased 11% ([7,15] *p* = .0003) with LPS, and 10% ([5,15] *p* = .003) with saline. SA decreased 36% ([48,21] *p* = .002) only with LPS. LA increased 40% ([16,70] *p* = .005) and AA increased 50% ([28,77] *p* = .0004) only with LPS. %AA was also lower at 15 min in the LPS group compared with the Saline group. %EPA is not reported because EPA was below the limit of detection. A baseline of 15 min was used because fatty acid concentrations could not be measured at 0 or 7.5 min due to low signal. ^*^Different from baseline (15 min perfusion) ^†^LPS different from Saline at baseline (15 min perfusion)

Next, we investigated the effect of LPS and sEHi treatment on fatty acid and oxylipin content of VLDL. The total amount of fatty acids in VLDL ultracentrifugation fraction increased gradually from 15 to 180 min, similar to fatty acid tracer enrichment within VLDL. Overall, LPS treatment decreased the total concentration of VLDL fatty acids across all time points by 30% [6%, 47%] (*p* = .02; Figure 3a). Rats treated with LPS had lower %AA than saline‐treated rats at 15 min (*p* < .05), suggesting that LPS treatment decreased the initial %AA in VLDL. %PA decreased from 15 min to 180 min in both treatments (*p* < .05). However, %SA, %LA, and %AA all changed with time only in the LPS group (%SA: LPS 15 min vs. 180 min, *p* = .002; Saline 15 min vs. 180 min, *p* = .2; %LA: LPS 15 min vs. 180 min, *p* = .005; Saline 15 min vs. 180 min, *p* = .8; Figure 3b%AA: LPS 15 min vs. 180 min, *p* = .0004; Saline 15 min vs. 180 min, *p* = .4). There was no effect of sEHi on VLDL fatty acid abundances.

LPS altered the final VLDL concentrations of several oxylipin species collected in the final perfusate (180 min), primarily derived from ω‐3 PUFA (ALA, EPA, and DHA; Table 1). The ALA‐derived alcohol, 13‐HOTrE, was 35% lower, and the ALA‐derived epoxides, 9(10)‐EpODE and 15(16)‐EpODE were both 29% lower with LPS treatment compared to saline (*p* < .05). However, 12(13)‐EpODE was not significantly affected by LPS. Two EPA epoxides, 14(15)‐EpETE and 17(18)‐EpETE, were quantified, but 17(18)‐EpETE, was uniquely 75% lower with LPS treatment compared to saline controls (*p* = .001). Only one DHA diol, 19(20)‐DiHDPA was reliably detected, and it was 31% lower with LPS treatment compared to saline (*p* = .03). AA diols (DiHETrEs) were the only ω‐6 oxylipins to be altered by LPS treatment, and they were 32% lower as a class with LPS treatment compared to saline (*p* = .046). LPS reduced most of these oxylipins by about 30%, similar to the LPS‐stimulated decrease in total VLDL fatty acids, but 17(18)‐EpETE was reduced by 75%, or greater than two times the amount of total fatty acid reduction in the VLDL fraction. sEHi altered concentrations of only one oxylipin, the DHA epoxide, 16(17)‐EpDPE, which was increased by 99% with sEHi (*p* = .005). We did not quantify other DHA epoxides.

**TABLE 1:** Effects of LPS and sEH inhibition (sEHi) on final oxylipin concentrations in VLDL

Chemistry	Oxylipin	Parent FA	Final Concentration at 180 min (nM, 95%CI)					
			LPS (*N* = 8)	Saline (*N* = 6)	*p*‐value^a^	sEHi (*N* = 7)	Vehicle (*N* = 7)	*p*‐value
Alcohols	9‐HODE	LA	191.1 (122.9, 296.9)	290.5 (174.6, 483.3)	NS	209.5 (130.4, 336.4)	264.9 (165.0, 425.5)	NS
	13‐HODE	LA	802.2 (516.2, 1,246.7)	1,203.4 (723.3, 2002.0)		874.4 (544.5, 1,404.2)	1,104.1 (687.5, 1773.1)	
	9‐HOTrE	ALA	2.5 (1.9, 3.3)	2.8 (2.1, 3.9)	NS	2.4 (1.8, 3.3)	2.9 (2.1, 3.8)	NS
	**13‐HOTrE**	**ALA**	**2.3 (1.8, 3.1)**	**3.6 (2.6, 4.9)**	**.04** *	2.9 (2.1, 3.8)	2.9 (2.2, 3.9)	
	5‐HETE	AA	69.8 (46.0, 105.8)	107.0 (66.2, 173.0)	NS	78.4 (50.1, 122.6)	95.3 (60.9, 149.0)	NS
	9‐HETE	AA	53.3 (35.1, 80.8)	76.1 (47.1, 123.1)		56.6 (36.2, 88.6)	71.6 (45.8, 112.0)	
	12‐HETE	AA	49.4 (32.6, 74.9)	72.4 (44.8, 117.1)		54.5 (34.8, 85.3)	65.6 (42.0, 102.7)	
	15‐HETE	AA	104.6 (69.0, 158.5)	175.3 (108.4, 283.5)		126.3 (80.8, 197.6)	145.1 (92.8, 227.0)	
	17‐HDoHE	DHA	7.4 (4.2, 13.0)	7.7 (4.0, 14.7)	NS	8.0 (4.4, 14.6)	7.1 (3.9, 13.1)	NS
Epoxides	9(10)‐EpOME	LA	110.0 (88.8, 136.2)	132.5 (103.5, 169.5)	NS	125.3 (99.6, 157.7)	116.2 (92.4, 146.3)	NS
	12(13)‐EpOME	LA	151.7 (122.5, 187.9)	182.4 (142.5, 233.5)		171.5 (136.3, 215.9)	161.3 (128.2, 203.0)	
	**9(10)‐EpODE**	**ALA**	**4.6 (3.7, 5.8)**	**6.6 (5.1, 8.5)**	**.05** *	5.8 (4.5, 7.3)	5.3 (4.2, 6.8)	NS
	12(13)‐EpODE	ALA	0.8 (0.7, 1.0)	1.0 (0.8, 1.3)	NS	1.0 (0.8, 1.3)	0.8 (0.7, 1.1)	
	**15(16)‐EpODE**	**ALA**	**3.7 (3.0, 4.7)**	**5.3 (4.1, 6.8)**	**.05** *	4.7 (3.7, 6.0)	4.2 (3.3, 5.3)	
	8(9)‐EpETrE	AA	38.6 (30.7, 48.4)	49.4 (38.0, 64.3)	NS	46.7 (36.5, 59.7)	40.8 (31.9, 52.1)	NS
	11(12)‐EpETrE	AA	85.7 (68.2, 107.6)	106.7 (82.0, 138.9)		100.1 (78.3, 128.0)	91.3 (71.4, 116.7)	
	14(15)‐EpETrE	AA	66.4 (52.9, 83.5)	87.8 (67.4, 114.3)		83.4 (65.2, 106.6)	69.9 (54.7, 89.4)	
	14(15)‐EpETE	EPA	0.60 (0.36, 0.98)	0.73 (0.41, 1.29)	NS	0.64 (0.38, 1.11)	0.67 (0.39, 1.14)	NS
	**17(18)‐EpETE**	**EPA**	**0.17 (0.10, 0.28)**	**0.69 (0.39, 1.24)**	**.001** **	0.29 (0.17, 0.50)	0.41 (0.24, 0.70)	
	**16(17)‐EpDPE**	**DHA**	2.9 (2.1, 3.8)	2.8 (2.0, 3.9)	NS	**4.0 (3.0, 5.4)**	**2.0 (1.5, 2.7)**	**.005** **
Diols	9(10)‐DiHOME	LA	19.3 (16.7, 22.3)	20.4 (17.2, 24.1)	NS	19.7 (16.9, 23.0)	20.0 (17.1, 23.3)	NS
	12(13)‐DiHOME	LA	3.8 (3.3, 4.4)	4.1 (3.5, 4.9)		3.8 (3.2, 4.4)	4.2 (3.6, 4.9)	
	9(10)‐DiHODE	ALA	0.08 (0.05, 0.13)	0.07 (0.04, 0.12)	NS	0.07 (0.04, 0.11)	0.09 (0.06, 0.15)	NS
	**5(6)‐DiHETrE**	**AA**	**2.2 (1.7, 2.9)**	**3.8 (2.7, 5.2)**	**.05** *	2.9 (2.2, 4.0)	2.8 (2.1, 3.7)	NS
	**8(9)‐DiHETrE**	**AA**	**0.5 (0.4, 0.7)**	**0.7 (0.5, 1.0)**		0.6 (0.4, 0.8)	0.7 (0.5, 0.9)	
	**11(12)‐DiHETrE**	**AA**	**0.7 (0.5, 0.9)**	**1.1 (0.8, 1.5)**		0.9 (0.7, 1.2)	0.8 (0.6, 1.1)	
	**14(15)‐DiHETrE**	**AA**	**0.6 (0.4, 0.8)**	**0.8 (0.6, 1.1)**		0.7 (0.5, 0.9)	0.7 (0.5, 1.0)	
	14(15)‐DiHETE	EPA	0.38 (0.30, 0.48)	0.46 (0.35, 0.60)	NS	0.45 (0.35, 0.58)	0.38 (0.30, 0.49)	NS
	17(18)‐DiHETE	EPA	2.5 (2.0, 3.1)	2.5 (1.9, 3.3)		2.3 (1.8, 2.9)	2.8 (2.2, 3.6)	
	**19(20)‐DiHDPA**	**DHA**	**0.21 (0.17, 0.27)**	**0.31 (0.24, 0.40)**	**.03** *	0.26 (0.20, 0.33)	0.26 (0.20, 0.33)	NS

^a^ Differences were assessed using 3‐way ANOVA to measure differences by LPS, sEHi, and regioisomer.

* *p *< .05.

** *p* < .01. Bold results are significantly different by treatment (*p* < .05).

**FIGURE 4:**
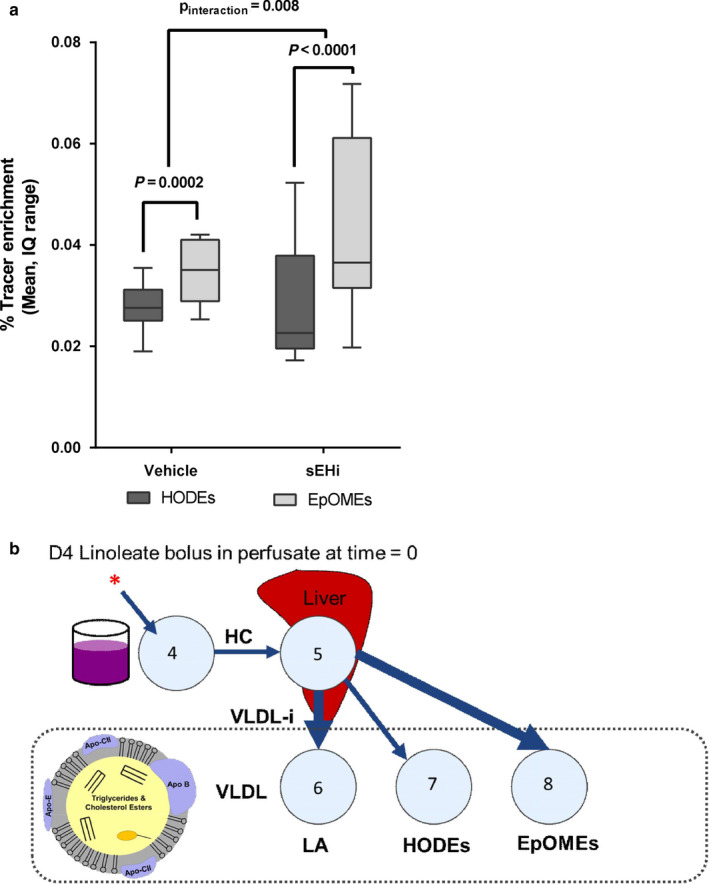
Effect of treatment on VLDL oxylipins. (a) sEH inhibition (sEHi) increased the proportion of tracer in LA epoxides (*p*_interaction_ = .008). LPS had no effect on tracer enrichment. (b) Theoretical model depicting how LA HC is related to the VLDL incorporation (VLDL‐i) of LA and LA‐oxylipins. Thicker arrows indicate higher incorporation, as measured by tracer enrichment. Although this model is based on the compartmental model in Figure 2, this is a theoretical depiction of the relationship to VLDL oxylipin concentrations, so transfer coefficients were not tested

We assessed the effect of sEHi on tracer enrichments in a subset of oxylipins with detectable label at 180 min. Tracer enrichment was higher in LA‐derived epoxides (EpOMEs) compared with LA‐derived mid chain alcohols (HODEs), and sEHi amplified this effect (Figure 4a) consistent with a decreased rate of epoxide hydrolysis. There was no observed effect of sEHi on tracer enrichment in 12‐HETE and LPS had no effect on tracer enrichment on any of detectable oxylipins in this experiments. A theoretical model depicting how HC of LA is related to the VLDL incorporation (VLDL‐i) of LA and LA‐oxylipins is shown in Figure 4b.

**FIGURE 5:**
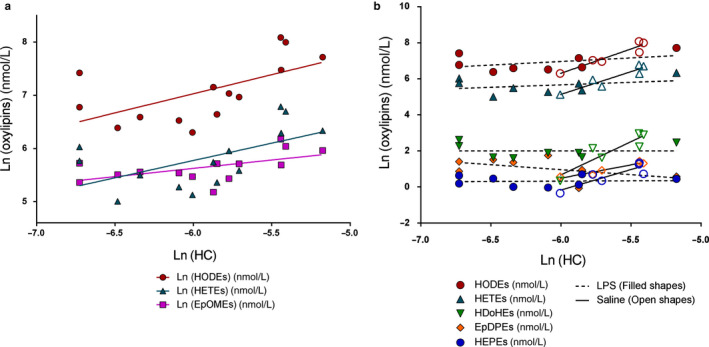
Correlations of final oxylipin composition with kinetic parameters. (a) Concentrations of several ω‐6 oxylipins in VLDL correlated with hepatic clearance (HC) by univariate regression analysis (HODE, *p* = .02, *r^2^* = .37; HETE, *p* = .03, *r* ^2^ = .33; EpOME, *p* = .03, *r* ^2^ = .32). No oxylipin classes were correlated with VLDL incorporation rate (VLDL‐i; Table S2). (b) ω‐6 alcohol correlations with HC were reduced with LPS (HODE, LPS *r* ^2^ = .19, Saline *r* ^2^ = .90, *p*_interaction_ = .015; HETE, LPS *r* ^2^ = .12, Saline *r* ^2^ = .87, *p*_interaction_ = .014). ω‐3 oxylipin concentrations were only correlated with HC when not treated with LPS (HEPE, LPS *r* ^2^ = .00, Saline *r* ^2^ = .77, *p*_interaction_ = .006; HDoHE, LPS *r* ^2^ = .00, Saline *r* ^2^ = .83, *p*_interaction_ = .002; EpDPE, LPS *r* ^2^ = .24, Saline *r* ^2^ = .93, *p*_interaction_ = .043). Soluble epoxide hydrolase inhibitor (sEHi) did not significantly affect correlations with kinetic parameters

It is expected that the final observed oxylipin concentrations in VLDL would be dependent on kinetic parameters describing PUFA (LA) incorporation into VLDL. We observed a significant overall association between hepatic LA clearance (HC) and the final concentration of oxylipins in VLDL (Figure 5a). No significant correlations were found with the LA VLDL incorporation parameter (VLDL‐i, Table S2), suggesting that primary regulation of oxylipin concentration occurs by regulation of PUFA uptake. The next question was whether inflammation affect the kinetics of oxylipins in VLDL. If LPS treatment alters regulation of oxylipin incorporation independent of fatty acid incorporation, the association between HC and final VLDL oxylipins will be reduced. To conduct this analysis with the maximum number of samples, we pooled the data from sEHi treated and untreated livers because sEHi had no effect on the association between kinetic parameters and oxylipin concentrations.

Under basal conditions (saline), there was a strong association of VLDL oxylipin concentration with the LA HC rate constant. Those rats with a higher HC had greater VLDL oxylipin abundance of nearly all mid‐chain alcohols and the DHA epoxide, 16(17)‐EpDPE. Rats treated with LPS had very low, or no association between HC and VLDL oxylipin concentrations (Figure 5b).

## 4 DISCUSSION

The detection of label in VLDL‐oxylipins, derived from nonesterified LA, is strong evidence that the liver utilizes circulating PUFAs for synthesis of oxylipins and incorporates them into VLDL. Additionally, the presence of label in AA‐derived 12‐HETE indicates that labeled LA was both elongated to AA and converted to 12‐HETE in isolated perfused rat livers, although this result should be interpreted with caution because 12‐HETE tracer was not detected above noise for all rats (Figure S1).

LPS decreases the uptake of LA by the liver, which corresponds with an approximately 30% decrease in several oxylipins in VLDL. One significant exception to this was 17(18)‐EpETE, an anti‐inflammatory EPA‐derived epoxide (Gabbs et al., 2015; Morin, Sirois, Échavé, Albadine, & Rousseau, 2009), which was uniquely decreased by 75%. This could indicate that the CYP450 epoxygenase pathway action on EPA is suppressed during the acute inflammatory response. LPS also decreased the total fatty acid content of the VLDL particle by about 30%, which paralleled the significant decrease seen in several oxylipins. This is different than the findings of other studies, which showed increased VLDL synthesis with LPS challenge in fasted rats (Aspichueta, Pérez‐Agote, Pérez, Ochoa, & Fresnedo, 2006; Bartolomé et al., 2012). However, in these previous studies, synthesis of apoB increased much more than TG synthesis. It is probable that in the intact animal, increased adipocyte lipolysis (Mehta et al., 2010) and increased nonesterified fatty acid flux to the liver are a necessary component for increased VLDL‐TG synthesis. LPS could still be increasing apoB synthesis in our model, suggesting the VLDL in our model are less lipid rich.

Inhibition of sEH affected VLDL oxylipin content to a lesser degree than LPS by increasing the concentration of the DHA‐derived epoxide, 16(17)‐EpDPE. We expected that the most pronounced changes would be observed in ω‐6 PUFA‐derived oxylipins, but, surprisingly, ω‐3 PUFA‐derived oxylipins appeared to be the primary molecules altered by treatment. Although there was a strong relationship between LA kinetic model parameters and VLDL oxylipin concentrations, LPS altered this relationship, presumably shifting toward more hepatic regulation. This is an important finding, since it shows that processes internal to the liver have a regulatory effect on the oxylipins that are incorporated into VLDL and have the potential to effect the peripheral tissues. Similarly, the preferential incorporation of labeled LA into epoxides compared to alcohols suggests different loading rates into VLDL.

There has been interest in using the CYP450 epoxygenase pathway as a therapeutic target to increase epoxide concentrations and optimize their potent anti‐inflammatory properties by sEHi (Duflot et al., (2014)). We have shown that VLDL are particularly important carriers of PUFA‐derived epoxides (Newman et al., 2007), and increasing ω‐3 PUFA intake preferentially increases VLDL epoxides (Newman et al., 2014). Additionally, VLDL epoxides are labile to LpL activity (Shearer & Newman, 2008) and therefore available to LpL‐expressing tissues, as well as cells which actively endocytose these particles via the VLDL receptor (Takahashi, Kawarabayasi, Nakai, Sakai, & Yamamoto, 1992).

In this experiment, we used the sEH inhibitor, AUDA, in the liver perfusates to examine this question. Unexpectedly, significant changes in VLDL epoxide concentrations with sEHi were only observed in the DHA‐derived 16(17)‐EpDPE, which nearly doubled. This may suggest a selective action on of sEHi on DHA‐derived epoxides. Tracer analysis showed that sEHi also increased the preferential tracer enrichment in EpOMEs relative to HODEs, which is consistent with sEHi slowing the hydrolysis of EpOMEs to diols.

If concentrations of oxylipins in VLDL are only based on the available substrate taken up by the liver, then the kinetic parameters for LA should correlate strongly with final VLDL‐oxylipin concentrations. Although several oxylipin classes were strongly correlated with LA HC in the saline condition, they were weakly or not at all correlated in the inflammatory condition (LPS). This lack of correlation with multi‐compartmental model parameters indicates that the liver actively regulates the synthesis and incorporation rates of oxylipins into VLDL during inflammation. It is unclear why oxylipin concentrations were not correlated with VLDL‐i; it is possible that, although both parameters were necessary to fit the data well, the HC parameter captured the majority of the important variance in the data.

Although we chose to trace ω‐6 fatty acids, with the exception of a small change in AA‐derived DiHETrEs, only ω‐3 oxylipin concentrations changed with LPS treatment. This suggests that the liver is more active in regulating the ω‐3 oxylipin incorporation into VLDL than ω‐6 oxylipins under these conditions. Another possibility is that the AA‐derived oxylipin pool is much larger, and small changes are more difficult to detect, requiring a larger sample size. Human studies have found that ω‐3 treatment increases circulating oxylipin products of ω‐3 PUFAs, and decreases AA‐derived oxylipins, especially in the VLDL (Akintoye et al., 2016; Keenan et al., 2012; Newman et al., 2014; Schebb et al., 2014; Schuchardt et al., 2014), supporting active hepatic regulation of ω‐3 metabolism. One notable exception is subjects with low baseline levels of AA, who show an increase in both ω‐3 and ω‐6 circulating oxylipins when treated with ω‐3 PUFA (Keenan et al., 2012). Since ω‐3 and ω‐6 oxylipins display diverse biological activities in multiple tissues, this implicates VLDL as a potential carrier for systemic oxylipin signals.

In addition, we observed that EPA‐ and DHA‐derived epoxides respond uniquely. Specifically, LPS decreased the EPA‐derived 17(18)‐EpETE more than any other oxylipin. In contrast, only the DHA‐derived 16(17)‐EpDPE was increased in response to sEHi. These differential epoxide responses may help to elucidate the unique individual effects of EPA and DHA. Comparative supplementation trials have shown that EPA and DHA both lower plasma triglycerides and reduce oxidative stress biomarkers (Mori, Puddey, et al., 2000), but may have unique effects on other plasma lipids, such as LDL‐C and HDL‐C (Allaire et al., 2016; Innes & Calder, 2018; Mori, Burke, et al., 2000). Distinct effects are increasingly important to understand with findings of profound cardioprotective effects of high dose EPA in prevention of primary and secondary cardiovascular events (Bhatt et al., 2018). Whether high dose treatments including DHA have similar reductions in risk is less clear, since recent trials using both EPA and DHA as fish oil have had mixed results (Abdelhamid et al., 2018; Bowman et al., 2018; Manson et al., 2018), and a large‐scale study using high dose DHA has not been conducted. Future studies should consider tracing EPA and DHA to determine the effects of pro‐ and anti‐inflammatory treatments on ω‐3 oxylipins.

### 4.1 Strengths and limitations

One of the most important strengths of this study was the use of the perfused liver as an isolated system, which control for the influence of other tissues on VLDL incorporation. The use of both the LPS challenge and a chemical inhibitor of the metabolic pathway in a factorial treatment design provides data on oxylipin regulation by manipulation of inflammatory condition and metabolic pathways. Another strength was the large number of oxylipins measured, which increased our ability to find treatment effects.

This study also had some important limitations. One was that we only used labeled LA in our experiment, providing tracer data for only the ω‐6, and not ω‐3, oxylipins. Another limitation was the small sample size of 8 rats per treatment group (4 per 2 × 2 group), which limits our power to detect group differences, however using an exploratory *p*‐value of <.1 did not change our conclusions, which suggests we can have reasonable confidence in our positive findings.

## 5 CONCLUSIONS

In conclusion, this study shows that the liver is active in regulating oxylipin synthesis and incorporation into VLDL. The incorporation of VLDL oxylipins can be altered by both inflammatory and pharmaceutical challenges. This suggests that the liver regulates the composition of VLDL in response to its pro‐ or anti‐inflammatory state, which may affect the oxylipin signals that are delivered systemically under various conditions.

## CONFLICT OF INTEREST

None declared.

## AUTHOR CONTRIBUTIONS

REW performed fatty acid analysis, compartmental modeling, and statistical analysis and wrote the manuscript. OVS performed the perfusion experiment and reviewed the manuscript. TLP and JWN performed the oxylipin analysis and reviewed the manuscript. GCS was responsible for overseeing the design, implementation, and analysis of the project and reviewed/edited the manuscript.

## Supporting information



Supplementary Material

## References

[phy214480-bib-0001] Abdelhamid, A. , Brown, T. , Brainard, J. , Biswas, P. , Thorpe, G. , Moore, H. , … Hooper, L.(2018). Omega 3 fatty acids for the primary and secondary prevention of cardiovascular disease. Cochrane Database of Systematic Reviews, (11), CD003177 10.1002/14651858.CD003177.pub4 PMC6517311 30521670

[phy214480-bib-0002] Agrawal, K. , Hassoun, L. A. , Foolad, N. , Pedersen, T. L. , & Sivamani, R. K. (2017). Sweat lipid mediator profiling: A noninvasive approach for cutaneous research. Journal of Lipid Research, 58, 188–195. 27875258 10.1194/jlr.M071738 PMC5234720

[phy214480-bib-0003] Akintoye, E. , Wu, J. H. Y. , Hou, T. , Song, X. , Yang, J. , Hammock, B. , & Mozaffarian, D. (2016). Effect of fish oil on monoepoxides derived from fatty acids during cardiac surgery. Journal of Lipid Research, 57(3), 492–498. 10.1194/jlr.P062398 26749073 PMC4766998

[phy214480-bib-0004] Allaire, J. , Couture, P. , Leclerc, M. , Charest, A. , Marin, J. , Marie‐claude, L. , … Tchernof, A. (2016). A randomized, crossover, head‐to‐head comparison of eicosapentaenoic acid and docosahexaenoic acid supplementation to reduce inflammation markers in men and women: The Comparing EPA to DHA (ComparED ). The American Journal of Clinical Nutrition, 104, 280–287. 27281302 10.3945/ajcn.116.131896

[phy214480-bib-0005] Aspichueta, P. , Pérez‐Agote, B. , Pérez, S. , Ochoa, B. , & Fresnedo, O. (2006). Impaired response of VLDL lipid and apoB secretion to endotoxin in the fasted rat liver. Journal of Endotoxin Research, 12(3), 181–192. 10.1179/096805106X102174 16719989

[phy214480-bib-0006] Bartolomé, N. , Aspichueta, P. , Martínez, M. J. , Vázquez‐Chantada, M. , Martínez‐Chantar, M. L. , Ochoa, B. , & Chico, Y. (2012). Biphasic adaptative responses in VLDL metabolism and lipoprotein homeostasis during Gram‐negative endotoxemia. Innate Immunity, 18(1), 89–99. 10.1177/1753425910390722 21113081

[phy214480-bib-0007] Bhatt, D. L. , Steg, P. G. , Miller, M. , Brinton, E. A. , Jacobson, T. A. , Ketchum, S. B. , … Ballantyne, C. M. (2018). Cardiovascular risk reduction with icosapent ethyl for hypertriglyceridemia. New England Journal of Medicine, 380(1), 11–22. 10.1056/NEJMoa1812792 30415628

[phy214480-bib-0008] Bligh, E. G. , & Dyer, W. J. (1959). A rapid method of total lipid extraction and purification. Canadian Journal of Biochemistry and Physiology, 37, 911–917. 10.1139/y59-099 13671378

[phy214480-bib-0009] Bowman, L. , Mafham, M. , Stevens, W. , Buck, G. , Barton, J. , Murphy, K. , … Armitage, J. (2018). Effects of n−3 fatty acid supplements in diabetes mellitus. The New England Journal of Medicine, 379, 1540–1550. 30146932 10.1056/NEJMoa1804989

[phy214480-bib-0010] Buczynski, M. W. , Dumlao, D. S. , & Dennis, E. A. (2009). An integrated omics analysis of eicosanoid biology 1. Journal of Lipid Research, 50, 1015–1038. 19244215 10.1194/jlr.R900004-JLR200 PMC2681385

[phy214480-bib-0011] Duflot, T. , Roche, C. , Lamoureux, F. , Guerrot, D. , & Bellien, J. (2014). Design and discovery of soluble epoxide hydrolase inhibitors for the treatment of cardiovascular diseases. Expert Opinion on Drug Discovery, 9(3), 229–243. 10.1517/17460441.2014.881354 24490654

[phy214480-bib-0012] Edelstein, C. , & Scanu, A. (1986). Precautionary measures for collecting blood destined for lipoprotein isolation. Methods in Enzymology, 128, 151–155. 3724499 10.1016/0076-6879(86)28065-9

[phy214480-bib-0013] Gabbs, M. , Leng, S. , Devassy, J. G. , Monirujjaman, M. , & Aukema, H. M. (2015). Advances in our understanding of oxylipins derived from dietary PUFAs. Advances in Nutrition, 6(5), 513–540. 10.3945/an.114.007732 26374175 PMC4561827

[phy214480-bib-0014] Gong, Y. , Fu, Z. , Edin, M. L. , Liu, C. H. , Wang, Z. , Shao, Z. , … Smith, L. E. H. (2016). Cytochrome P450 oxidase 2C inhibition adds to ω‐3 long‐chain polyunsaturated fatty acids protection against retinal and choroidal neovascularization. Arteriosclerosis, Thrombosis, and Vascular Biology, 36(9), 1919–1927. 10.1161/ATVBAHA.116.307558 PMC5010176 27417579

[phy214480-bib-0015] Grapov, D. , Adams, S. H. , Pedersen, T. L. , Garvey, W. T. , & Newman, J. W. (2012). Type 2 diabetes associated changes in the plasma non‐esterified fatty acids, oxylipins and endocannabinoids. PLoS One, 7, 1–11. 10.1371/journal.pone.0048852 PMC3493609 23144998

[phy214480-bib-0016] Innes, J. K. , & Calder, P. C. (2018). The differential effects of eicosapentaenoic acid and docosahexaenoic acid on cardiometabolic risk factors: A systematic review. International Journal of Molecular Sciences, 19(2), 532– 10.3390/ijms19020532 PMC5855754 29425187

[phy214480-bib-0017] Keenan, A. H. , Pedersen, T. L. , Fillaus, K. , Larson, M. K. , Shearer, G. C. , & Newman, J. W. (2012). Basal omega‐3 fatty acid status affects fatty acid and oxylipin responses to high‐dose n3‐HUFA in healthy volunteers. Journal of Lipid Research, 53(8), 1662–1669. 10.1194/jlr.P025577 22628615 PMC3540841

[phy214480-bib-0018] Magnifico, M. C. , Oberkersch, R. E. , Mollo, A. , Giambelli, L. , Grooten, Y. , Sarti, P. , … Arese, M. (2017). VLDL induced modulation of nitric oxide signalling and cell redox homeostasis in HUVEC. Oxidative Medicine and Cellular Longevity, 2017, 1–15. 10.1155/2017/2697364 PMC5632467 29085553

[phy214480-bib-0019] Manson, J. E. , Cook, N. R. , Lee, I.‐M. , Christen, W. , Bassuk, S. S. , Mora, S. , … Buring, J. E. (2018). Marine n−3 fatty acids and prevention of cardiovascular disease and cancer. New England Journal of Medicine, 380(1), 23–32. 10.1056/NEJMoa1811403 PMC6392053 30415637

[phy214480-bib-0020] Markworth, J. F. , Kaur, G. , Miller, E. G. , Larsen, A. E. , Sinclair, A. J. , Maddipati, K. R. , & Cameron‐Smith, D. (2016). Divergent shifts in lipid mediator profile following supplementation with n‐3 docosapentaenoic acid and eicosapentaenoic acid. The FASEB Journal, 30(11), 3714–3725. 10.1096/fj.201600360R 27461565 PMC5067251

[phy214480-bib-0021] Mehta, N. N. , McGillicuddy, F. C. , Anderson, P. D. , Hinkle, C. C. , Shah, R. , Pruscino, L. , … Reilly, M. P. (2010). Experimental endotoxemia induces adipose inflammation and insulin resistance in humans. Diabetes, 59(1), 172–181. 10.2337/db09-0367 19794059 PMC2797919

[phy214480-bib-0022] Mori, T. A. , Burke, V. , Puddey, I. B. , Watts, G. F. , Neal, D. N. O. , Best, J. D. , & Beilin, L. J. (2000). Purified eicosapentaenoic and docosahexaenoic acids have differential effects on serum lipids and lipoproteins, LDL particle size, glucose, and insulin in mildly hyperlipidemic men 1–3. The American Journal of Clinical Nutrition, 71(5), 1085–1094. 10.1093/ajcn/71.5.1085 10799369

[phy214480-bib-0023] Mori, T. A. , Puddey, I. B. , Burke, V. , Croft, K. D. , Dunstan, D. W. , Rivera, J. H. , … Beilin, L. J. (2000). Effect of ω 3 fatty acids on oxidative stress in humans : GC – MS measurement of urinary F 2 ‐ isoprostane excretion short refereed paper effect of ω 3 fatty acids on oxidative stress in humans : GCÐMS measurement o. Redox Report, 5, 45–46. 10905544 10.1179/rer.2000.5.1.45

[phy214480-bib-0024] Morin, C. , Sirois, M. , Échavé, V. , Albadine, R. , & Rousseau, E. (2009). 17,18‐Epoxyeicosatetraenoic acid targets PPARγ and p38 mitogen‐activated protein kinase to mediate its anti‐inflammatory effects in the lung. American Journal of Respiratory Cell and Molecular Biology, 43(5), 564–575. 10.1165/rcmb.2009-0155OC 20008283

[phy214480-bib-0025] Morisseau, C. , & Hammock, B. D. (2012). Impact of Soluble Epoxide Hydrolase and Epoxyeicosanoids on Human Health. Annual Review of Pharmacology and Toxicology, 53(1), 37–58. 10.1146/annurev-pharmtox-011112-140244 PMC3578707 23020295

[phy214480-bib-0026] Morrison, W. R. , & Smith, L. M. (1964). Preparation of fatty acid methyl esters and dimethylacetals from lipids with boron fluoride‐methanol. Journal of Lipid Research, 5, 600–608. 14221106

[phy214480-bib-0027] Newman, J. W. , Kaysen, G. A. , Hammock, B. D. , & Shearer, G. C. (2007). Proteinuria increases oxylipid concentrations in VLDL and HDL but not LDL particles in the rat. Journal of Lipid Research, 48(8), 1792–1800. 10.1194/jlr.M700146-JLR200 17496268

[phy214480-bib-0028] Newman, J. W. , Pedersen, T. L. , Brandenburg, V. R. , Harris, W. S. , & Shearer, G. C. (2014). Effect of omega‐3 fatty acid ethyl esters on the oxylipin composition of lipoproteins in hypertriglyceridemic, statin‐treated subjects. PLoS One, 9, 1–12. 10.1371/journal.pone.0111471 PMC4230929 25393536

[phy214480-bib-0029] Oni‐orisan, A. , Edin, M. L. , Lee, J. A. , Wells, M. A. , Christensen, E. S. , Vendrov, K. C. , … Lee, C. R. (2016). Cytochrome P450‐derived epoxyeicosatrienoic acids and coronary artery disease in humans : A targeted metabolomics study. Journal of Lipid Research, 57, 109–119. 26555503 10.1194/jlr.M061697 PMC4689337

[phy214480-bib-0030] Proudfoot, J. M. , Barden, A. E. , Loke, W. M. , Croft, K. D. , Puddey, I. B. , & Mori, T. A. (2009). HDL is the major lipoprotein carrier of plasma F 2 ‐isoprostanes. Journal of Lipid Research, 50, 716–722. 19050315 10.1194/jlr.M800607-JLR200 PMC2656665

[phy214480-bib-0031] Schebb, N. H. , Ostermann, A. I. , Yang, J. , Hammock, B. D. , Hahn, A. , & Philipp, J. (2014). Comparison of the effects of long‐chain omega‐3 fatty acid supplementation on plasma levels of free and esterified oxylipins. Prostaglandins & Other Lipid Mediators, 113, 21–29. 10.1016/j.prostaglandins.2014.05.002 24880049 PMC4247815

[phy214480-bib-0032] Schuchardt, J. P. , Schneider, I. , Willenberg, I. , Yang, J. , Hammock, B. D. , Hahn, A. , & Schebb, N. H. (2014). Increase of EPA‐derived hydroxy, epoxy and dihydroxy fatty acid levels in human plasma after a single dose of long‐chain omega‐3 PUFA. Prostaglandins & Other Lipid Mediators, 109, 23–31. 10.1016/j.prostaglandins.2014.03.001 24667634 PMC4172375

[phy214480-bib-0033] Shao, Z. , Fu, Z. , Stahl, A. , Joyal, J. S. , Hatton, C. , Juan, A. , … Smith, L. E. H. (2014). Cytochrome P450 2C8 ω3‐long‐chain polyunsaturated fatty acid metabolites increase mouse retinal pathologic neovascularization‐brief report. Arteriosclerosis, Thrombosis, and Vascular Biology, 34(3), 581–586. 10.1161/ATVBAHA.113.302927 PMC4005334 24458713

[phy214480-bib-0034] Shearer, G. C. , Borkowski, K. , Puumala, S. L. , Harris, W. S. , Pedersen, T. L. , & Newman, J. W. (2018). Abnormal lipoprotein oxylipins in metabolic syndrome and partial correction by omega‐3 fatty acids. Prostaglandins, Leukotrienes and Essential Fatty Acids, 28, 1–10. 10.1016/j.plefa.2017.10.006 29413356

[phy214480-bib-0035] Shearer, G. C. , Couser, W. G. , & Kaysen, G. A. (2004). Nephrotic livers secrete normal VLDL that acquire structural and functional defects following interaction with HDL. Kidney International, 65(1), 228–237. 10.1111/j.1523-1755.2004.00373.x 14675054

[phy214480-bib-0036] Shearer, G. C. , Harris, W. S. , Pedersen, T. L. , & Newman, J. W. (2010). Detection of omega‐3 oxylipins in human plasma and response to treatment with omega‐3 acid ethyl esters. Journal of Lipid Research, 51(8), 2074–2081. 10.1194/jlr.M900193-JLR200 19671931 PMC2903824

[phy214480-bib-0037] Shearer, G. C. , & Newman, J. W. (2008). Lipoprotein lipase releases esterified oxylipins from very low‐density lipoproteins. Prostaglandins, Leukotrienes and Essential Fatty Acids, 79(6), 215–222. 10.1016/j.plefa.2008.09.023 PMC2629508 19042114

[phy214480-bib-0038] Shearer, G. C. , & Newman, J. W. (2009). Impact of circulating esterified eicosanoids and other oxylipins on endothelial function. Current Atherosclerosis Reports, 11(6), 403–410. 10.1007/s11883-009-0061-3 19852880

[phy214480-bib-0039] Shearer, G. C. , & Walker, R. E. (2018). An overview of the biologic effects of omega‐6 oxylipins in humans. Prostaglandins, Leukotrienes and Essential Fatty Acids, 137, 26–38. 10.1016/j.plefa.2018.06.005 30293594

[phy214480-bib-0040] Smedes, F. (1999). Determination of total lipid using non‐chlorinated solvents. The Analyst, 124(11), 1711–1718. 10.1039/a905904k

[phy214480-bib-0041] Spector, A. A. (2009). Arachidonic acid cytochrome P450 epoxygenase pathway. Journal of Lipid Research, 50(Supplement), S52–S56. 10.1194/jlr.R800038-JLR200 18952572 PMC2674692

[phy214480-bib-0042] Spector, A. A. , & Kim, H. Y. (2015). Cytochrome P epoxygenase pathway of polyunsaturated fatty acid metabolism. Biochimica Et Biophysica Acta, 1851, 356–365. 25093613 10.1016/j.bbalip.2014.07.020 PMC4314516

[phy214480-bib-0043] Spector, A. A. , & Norris, A. W. (2007). Action of epoxyeicosatrienoic acids on cellular function. American Journal of Physiology‐Cell Physiology, 292(3), C996–C1012. 10.1152/ajpcell.00402.2006 16987999

[phy214480-bib-0044] Takahashi, S. , Kawarabayasi, Y. , Nakai, T. , Sakai, J. , & Yamamoto, T. (1992). Rabbit very low density lipoprotein receptor: A low density lipoprotein receptor‐like protein with distinct ligand specificity. Proceedings of the National Academy of Sciences of the United States of America, 89, 9252–9256. 1384047 10.1073/pnas.89.19.9252 PMC50104

[phy214480-bib-0045] Ulu, A. , Davis, B. B. , Tsai, H. , Kim, I. , Morisseau, C. , Inceoglu, B. , … Weiss, R. H. (2008). Soluble epoxide hydrolase inhibitors reduce the development of atherosclerosis in apolipoprotein E‐knockout mouse model. Journal of Cardiovascular Pharmacology, 52(4), 314–323. 10.1097/FJC.0b013e318185fa3c 18791465 PMC2637359

[phy214480-bib-0046] Wang, L. , Gill, R. , Pedersen, T. L. , Higgins, L. J. , Newman, J. W. , & Rutledge, J. C. (2009). Triglyceride‐rich lipoprotein lipolysis releases neutral and oxidized FFAs that induce endothelial cell inflammation. Journal of Lipid Research, 50(2), 204–213. 10.1194/jlr.M700505-JLR200 18812596 PMC2636918

[phy214480-bib-0047] Wang, L. , Sapuri‐Butti, A. R. , Aung, H. H. , Parikh, A. N. , & Rutledge, J. C. (2008). Triglyceride‐rich lipoprotein lipolysis increases aggregation of endothelial cell membrane microdomains and produces reactive oxygen species. American Journal of Physiology‐Heart and Circulatory Physiology, 295(1), H237–H244. 10.1152/ajpheart.01366.2007 18487440 PMC2494756

[phy214480-bib-0048] Wells, M. A. , Vendrov, K. C. , Edin, M. L. , Ferslew, B. C. , Zha, W. , Nguyen, B. K. H. , … Lee, C. R. (2016). Characterization of the Cytochrome P450 epoxyeicosanoid pathway in non‐alcoholic steatohepatitis. Prostaglandins & Other Lipid Mediators, 125, 19–29. 10.1016/j.prostaglandins.2016.07.002 27401401 PMC5035202

[phy214480-bib-0049] Yanai, R. , Mulki, L. , Hasegawa, E. , Takeuchi, K. , Sweigard, H. , Suzuki, J. , … Connor, K. M. (2014). Cytochrome P450‐generated metabolites derived from ω‐3 fatty acids attenuate neovascularization. Proceedings of the National Academy of Sciences, 111(26), 9603–9608. 10.1073/pnas.1401191111 PMC4084420 24979774

[phy214480-bib-0050] Ye, D. , Zhang, D. , Oltman, C. , Dellsperger, K. , Lee, H.‐C. , & VanRollins, M. (2002). Cytochrome p‐450 epoxygenase metabolites of docosahexaenoate potently dilate coronary arterioles by activating large‐conductance calcium‐activated potassium channels. Journal of Pharmacology and Experimental Therapeutics, 303(2), 768–776. 10.1124/jpet.303.2.768 12388664

