# Supplementary material for: Effects of inflammation and soluble epoxide hydrolase inhibition on oxylipin composition of very low‐density lipoproteins in isolated perfused rat livers
Source: Physiol Rep. 2021 Feb 24;9(4):e14480. doi: 10.14814/phy2.14480 (PMC7903942; doi:10.14814/phy2.14480)
Supplement: Supplementary file 1 — Supplementary Material [file PHY2-9-e14480-s001.docx]

**Supplemental Material**

Supplemental Table 1 provides details of the mass spectrometry detection settings. Specifically, the parent molecular ion and product ion m/z values are provided, along with retention times in minutes, source voltage, and collision energy for both the native and deuterium labeled oxylipin species. Supplemental Figure 1 and Figure 2 provide representative chromatograms for the native and labeled oxylipin mass transitions for the multireaction monitoring (MRM) analysis.

**Supplemental Table 1: Mass Transitions for MRM analysis of deuterated oxylipins**

| **Analyte and signal status** | | **Analyte LC-MS/MS acquisition parameters** | | | | |
| --- | --- | --- | --- | --- | --- | --- |
| **Analyte** | **Signal status** | **Molecular ion (Q1)** | **Product ion (Q3)** | **Retention time** | **Source voltage (DCP)** | **Collision energy (CE)** |
| PHAU -esi | yes | 249.2 | 130.1 | 3.06 | -30 | -18 |
| CUDA -esi | yes | 339.4 | 214.2 | 8.7 | -70 | -30 |
| 20-HETE | yes, small | 319.2 | 275.2 | 11.03 | -95 | -24 |
| d* 19-HETE | no | 323.2 | 279.2 | 11.03 | -95 | -24 |
| d* 20-HETE | no | 323.2 | 279.2 | 11.03 | -95 | -24 |
| 13-HODE | yes | 295.201 | 195.2 | 11.89 | -90 | -24 |
| d* 13-HODE | yes | 299.201 | 198.1 | 11.89 | -90 | -24 |
| 9-HODE | yes | 295.201 | 171.1 | 12.09 | -70 | -24 |
| d* 9-HODE | yes | 299.201 | 172.1 | 12.09 | -70 | -24 |
| 15-HETE | yes | 319.201 | 219.2 | 12.27 | -55 | -18 |
| d* 15-HETE | not above noise | 323.201 | 222.1 | 12.27 | -55 | -18 |
| 13-KODE | 2 co-eluters | 293.2 | 179.1 | 12.33 | -80 | -27 |
| d* 13-KODE | not above noise | 297.2 | 180.1 | 12.33 | -80 | -27 |
| 11-HETE | yes | 319.201 | 167.1 | 12.7 | -45 | -21 |
| d* 11-HETE | close to noise | 323.201 | 167.1 | 12.7 | -45 | -21 |
| 9-KODE | yes | 293.2 | 185.2 | 12.77 | -100 | -27 |
| d* 9-KODE | no | 297.2 | 185.2 | 12.77 | -100 | -27 |
| 12-HETE | yes | 319.2 | 179.1 | 12.92 | -60 | -21 |
| d* 12-HETE | yes, small | 323.2 | 180.1 | 12.92 | -60 | -21 |
| 8-HETE | yes | 319.201 | 155.1 | 13.05 | -45 | -21 |
| d* 8-HETE | not above noise | 323.201 | 155.1 | 13.05 | -45 | -21 |
| 9-HETE | yes | 319.202 | 167.1 | 13.22 | -60 | -18 |
| d* 9-HETE | yes, small | 323.202 | 167.1 | 13.22 | -60 | -18 |
| 5-HETE | yes | 319.2 | 115.1 | 13.54 | -50 | -18 |
| d* 5-HETE | not above noise | 323.2 | 115.1 | 13.54 | -50 | -18 |
| 12(13)-EpOME | yes | 295.202 | 195.2 | 13.67 | -85 | -21 |
| d* 12(13)-EpOME | yes | 299.202 | 198.1 | 13.67 | -85 | -21 |
| 14(15)-EpETrE | yes | 319.202 | 219.2 | 13.75 | -50 | -18 |
| d* 14(15)-EpETrE | not above noise | 323.202 | 222.1 | 13.75 | -50 | -18 |
| 9(10)-EpOME | yes | 295.202 | 171.1 | 13.88 | -75 | -21 |
| d* 9(10)-EpOME | yes | 299.202 | 172.1 | 13.88 | -75 | -21 |
| 11(12)-EpETrE | yes | 319.203 | 167.1 | 14.18 | -40 | -21 |
| d* 11(12)-EpETrE | not above noise | 323.203 | 167.1 | 14.18 | -40 | -21 |
| 8(9)-EpETrE | yes | 319.202 | 155.1 | 14.36 | -40 | -18 |
| d* 8(9)-EpETrE | no | 323.202 | 155.1 | 14.36 | -40 | -18 |
| Arachidonate | yes | 303.4 | 259.2 | 16.6 | -40 | -18 |
| d4 Arachidonate | yes | 307.4 | 263.2 | 16.6 | -40 | -18 |
| Linoleate | yes | 279.4 | 261.2 | 16.89 | -185 | -38 |
| d4 Linoleate | yes | 283.4 | 264.2 | 16.89 | -185 | -38 |

**Supplemental Table 2: Univariate correlations between VLDL oxylipins and model parameters**

| **Oxylipin Class** | **Parent Fatty Acid** | **Chemistry** | **HC Correlation (r^2^)** | **p-value** | **VLDL-i Correlation (r^2^)** | **p-value** |
| --- | --- | --- | --- | --- | --- | --- |
| HODEs | LA | ω-6 | **0.37** | **0.02** | 0.01 | 0.73 |
| HETEs | AA | ω-6 | **0.33** | **0.03** | 0.00 | 0.98 |
| HEPEs | EPA | ω-3 | 0.20 | 0.11 | 0.00 | 0.83 |
| HDoHEs | DHA | ω-3 | 0.07 | 0.37 | 0.09 | 0.29 |
| EpOMEs | LA | ω-6 | **0.23** | **0.03** | 0.03 | 0.52 |
| EpETrEs | AA | ω-6 | 0.23 | 0.08 | 0.00 | 0.94 |
| EpETEs | EPA | ω-3 | 0.04 | 0.51 | 0.00 | 0.94 |
| EpDPEs | DHA | ω-3 | 0.04 | 0.47 | 0.02 | 0.63 |


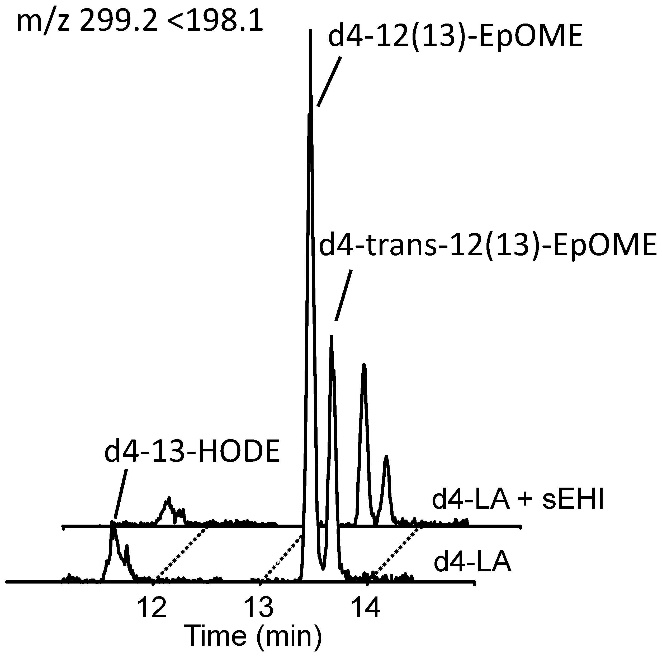


**Supplemental Figure 1: Representative chromatogram of perfused liver deuterated linoleate-derived epoxides in the presence or absence of soluble epoxide hydrolase inhibitor.** Deuterium containing compounds are putatively identified based on relative retention time to non-deuterated standards, or in the case of the trans-isomers, relative retention time to the identified compound. Shown are the d4‑13‑hydroxy-octadecanoic acid (13-HODE), d4‑12(13)‑epoxy-octadeca-9Z-eneoic acid and its putative d4‑trans-isomer, either 12(13)-epoxyoctadeca-9E-eneoic acid or trans-12(13)-epoxyoctadeca-9Z-eneoic acid. Chromatograms represent raw data area counts with minimal smoothing for integration.

**
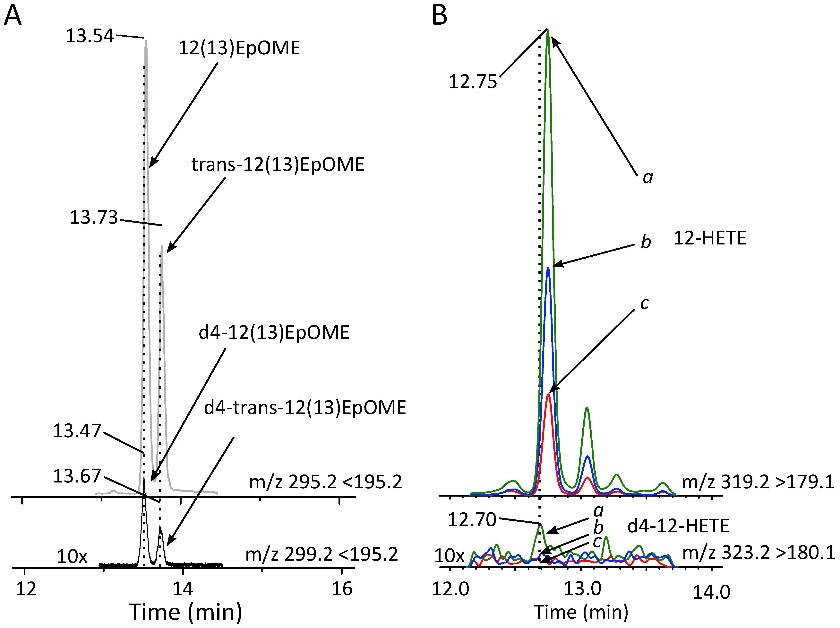
**

**Supplemental Figure 2: Representative chromatograms of deuterated linoleate-derived metabolites in VLDL isolated from perfused rat livers.** A) Traces for the native (upper) and deuterated (lower) forms of 12(13)-epoxyoctadeca-9Z-eneoic acid and its putative trans-isomer (either 12(13)-epoxyoctadeca-9E-eneoic acid or trans-12(13)-epoxyoctadeca-9Z-eneoic acid. B) Overlaid traces for 3 basal state rats (a, b, and c) for the native (upper) and deuterated (lower) forms of 12-HETE. Deuterium containing compounds are putatively identified based on relative retention time to non-deuterated standards, or in the case of the trans-isomers, relative retention time to the identified compound. Chromatograms represent raw data area counts with minimal smoothing for integration.
